# Function of Chick Subcutaneous Adipose Tissue During the Embryonic and Posthatch Period

**DOI:** 10.3389/fphys.2021.684426

**Published:** 2021-06-22

**Authors:** Haidong Zhao, Mingli Wu, Xiaoqin Tang, Qi Li, Xiaohua Yi, Shuhui Wang, Cunling Jia, Zehui Wei, Xiuzhu Sun

**Affiliations:** ^1^College of Animal Science and Technology, Northwest A&F University, Xianyang, China; ^2^College of Grassland Agriculture, Northwest A&F University, Xianyang, China

**Keywords:** subcutaneous adipose tissue, chicken, embryonic period, posthatch period, lipometabolism

## Abstract

Since excess abdominal fat is one of the main problems in the broiler industry for the development of modern broiler and layer industry, the importance of subcutaneous adipose tissue has been neglected. However, chick subcutaneous adipose tissue appeared earlier than abdominal adipose tissue and more than abdominal adipose tissue. Despite a wealth of data, detailed information is lacking about the development and function of chick subcutaneous adipose tissue during the embryonic and posthatch period. Therefore, the objective of the current study was to determine the developmental changes of adipocyte differentiation, lipid synthesis, lipolysis, fatty acid β-oxidation, and lipid contents from E12 to D9.5. The results showed that subcutaneous adipose tissue was another important energy supply tissue during the posthatch period. In this stage, the mitochondrial copy number and fatty acid β-oxidation level significantly increased. It revealed that chick subcutaneous adipose tissue not only has the function of energy supply by lipidolysis but also performs the same function as brown adipose tissue to some extent, despite that the brown adipose tissue does not exist in birds. In addition, this finding improved the theory of energy supply in the embryonic and posthatch period and might provide theoretical basis on physiological characteristics of lipid metabolism in chicks.

## Introduction

For a long period of time, the genetic breeding goal of meat-type chickens was to improve the growth rate, but the rapid growth of body weight is accompanied with the problem of excessive abdominal fat deposition (Moreira et al., 2018; Liu et al., 2020; Mohammadpour et al., 2020). For improve feed conversion rate and grain conservation, many researchers have studied the metabolic mechanism of abdominal fat deposition in chickens, ignoring the significance of subcutaneous adipose tissue (Simon and Leclercq, 1982; Na et al., 2018). Abdominal adipose tissue compared with subcutaneous adipose tissue is more cellular, vascular, innervated, and contains a larger number of inflammatory and immune cells, lesser preadipocyte differentiating capacity, and a greater percentage of large adipocytes, while subcutaneous adipose tissue is more avid in absorption of circulating free fatty acids and triglycerides (Ibrahim, 2010). The significance of subcutaneous fat deposition during the embryonic period is unknown. In the first few days after the hatching of chicks, yolk is rapidly absorbed. Therefore, we hypothesize that the function of subcutaneous adipose tissue may be associated with absorption of circulating lipid after hatching. The development of digestive system is not complete after hatching; extra energy supply could be an important function of subcutaneous fat deposition during the embryonic and posthatch period (Halevy, 2020; Lu et al., 2020; Nyuiadzi et al., 2020). Chick subcutaneous adipose tissue develops during the embryonic Day 12 and maintains a relatively stable content throughout the lifetime. In contrast, chick abdominal fat develops later than subcutaneous adipose tissue and has a strong relationship with feeding and management (Mellouk et al., 2018; Hicks and Liu, 2021).

During the past two decades, growth and development of white adipose tissue (WAT) and brown adipose tissue (BAT) have been extensively studied in various mammals and humans (Lu et al., 2021; Van Schaik et al., 2021). BAT evolved as a specialized thermogenic organ that is responsible for adaptive non-shivering thermogenesis (NST). For NST, energy metabolism of BAT mitochondria is increased by activation of uncoupling protein 1 (UCP1), which dissipates the proton motive force as heat. There was insufficient evidence to justify the presence of BAT and UCP1 gene in chickens (Mezentseva et al., 2008; Sotome et al., 2021). It is unclear whether subcutaneous adipose tissue is another important source of NST in chickens (Cristina-Silva et al., 2021). It is generally believed that chicken adipose tissue is similar to that of human beings, and neither has a strong ability of *de novo* fat synthesis. This is different from rodents, wherein both liver and adipose tissues have equal importance on *de novo* fat synthesis (Miska et al., 2015; Cogburn et al., 2020). From this point, chickens and humans have higher similarity in adipocyte lipid metabolism, and it is essential to understand the development and function of adipocyte lipid metabolism during the embryonic and posthatch periods. Despite a wealth of data, detailed information is lacking regarding the development of subcutaneous adipose tissue during embryogenesis and after hatching (Mellouk et al., 2018; Xiao et al., 2019).

Therefore, the objective of the current study was to determine the developmental changes of adipocyte differentiation, lipid synthesis, lipolysis, fatty acid β-oxidation, and lipid contents from E12 to D9.5 in chick subcutaneous adipose tissues, aiming to provide theoretical basis on physiological characteristics about lipid metabolism in chicks.

## Materials and Methods

All experimental procedures were performed in accordance with the Regulations for the Administration of Affairs Concerning Experimental Animals approved by the State Council of People’s Republic of China. The study was approved by the Institutional Animal Care and Use Committee of Northwest A&F University (Permit Number: NWAFAC1019).

### Sampling Collection

Lohmann pink chicken embryos and chicks used in the current study were bought from the Yangling Julong Poultry Industry Co. Ltd. (Yangling, China). Incubation conditions are as follows: E1∼E19, 37.8°C, E19∼E21, 37 37.5°C (Qingdao Xinyi Electronic Equipment Co., Ltd. Qingdao, China). Considering that the incubation stage of chicken embryo has certain individual differences, 10 eggs were selected randomly and taken out from the same incubator every 2 days from the embryonic day 12, marked E12, E14, E16, E18, and E20. After hatching, the chicks were allowed *ad libitum* access to water and feed. Considering that the chicks are not hatched at the same time, 10 chicks were selected randomly at the age of 1.5, 3.5, 5.5, 7.5, and 9.5 days after hatching (D1.5, D3.5, D5.5, D7.5, D9.5). Ten stages cover the appearance of subcutaneous adipose tissue to the disappearance of yolk. Chicken embryos and chicks were humanely euthanized by cervical dislocation and weighed after removing surface liquid with filter paper. Adipose, intestine, yolk, and liver tissues were rinsed thoroughly with ice-cold phosphate-buffered saline to remove blood contamination on the surface and also weighed for the tissues index calculation. Adipose samples were fixed in 4% formaldehyde for subsequent histological analysis using hematoxylin-eosin (HE) and immunohistochemistry (IHC). Tissues were collected in centrifuge tubes and stored at room temperature. Complete blood was collected in centrifuge tubes, and serum was harvested following centrifugation at 3,000 rpm at 4°C for 10 min until analysis from E20, D1.5, and D9.5. All the samples for RNA and protein analysis were stored at −80°C.

### HE and IHC Staining

After fixing, the adipose samples were processed through a series of procedures including dehydration, paraffin embedding, sectioning, and staining. All these procedures were performed by Wuhan Servicebio Technology Co., Ltd. (Wuhan, China). Primary antibody: pref-1 (Sangon, Shanghai, China). According to HE and IHC staining, adipocyte size, preadipocyte number, and cell proportion were calculated. The number of adipocytes per unit area represents the volume of adipocytes. The number of preadipocytes per unit area represents the preadipocyte number. The ratio of adipocyte number and preadipocyte number represents the cell proportion.

### Gene Expression

The deposition, decomposition, and utilization of fat are important processes in the function of adipose tissue. The maker genes of adipocyte differentiation (PPARγ, C/EBPα, and FABP4), lipid synthesis (ACC, FAS, and LPL), lipolysis (ATGL and HSL), and fatty acid β-oxidation (PPARα, CPT1A, and CPT2) are then measured from E12 to D9.5 chick adipose tissues. Total RNA extraction and cDNA synthesis from adipose and liver samples were performed according to reagent protocols using TRIzol and Primer Script RT Reagent kits (TaKaRa, Dalian, China). Relative gene expression was quantified by real-time quantitative PCR (qPCR). The assay was carried out *via* SYBR Premix Ex Taq kit (TaKaRa, Dalian, China) on the Y480 (Roche, Basel, Switzerland). Detailed reaction system was referred to our previous research (Zhao et al., 2020). Primer sequences used in the current study were all obtained from GenBank and shown in Table 1. All samples were run in triplicate, and the average cycle threshold (Ct) values were calculated for quantification using the 2^–ΔΔ^Ct method (Livak and Schmittgen, 2001).

**TABLE 1:** Primers list in this study.

Genes	Accession number	Sequencing (5′–3′)	Notes
*PPAR*γ	NM_001001460.1	F:AGTCCTTCCCGCTGACCAAA	qPCR
		R:TCTCCTGCACTGCCTCCACA	
*C/EBP*α	NM_001031459.1	F:TCCCACCTGCAGTACCAGAT	qPCR
		R:TTTTGGATTTGCCGCGGTG	
*A-FABP*	NM_204290.1	F:GCCAAGCCTAATTTAACTATCA	qPCR
		R:CAGCAGGTTCCCATCCAC	
*ACC*	NM_205505.1	F:GCTTCCCATTTGCCGTCCTA	qPCR
		R:GCCATTCTCACCACCTGATTACT	
*FASN*	NM_205155.2	F:TTTGGTGGTTCGAGGTGGTA	qPCR
		R:CAAAGGTTGTATTTCGGGAGC	
*LPL*	NM_205282.1	F:TTGGTGACCTGCTTATGCTA	qPCR
		R:TGCTGCCTCTTCTCCTTTAC	
*ATGL*	NM_001113291.1	F:TGCGTGGAGTGAGATATGTTGA	qPCR
		R:TTGCGAAGGTTGAATTGGAT	
*HSL*	XM_025155301.1	F:GTCTCGGGTTCCAGTTCGTG	qPCR
		R:CGTAGGACACCAACCCGATG	
*PPAR*α	NM_001001464.1	F:CAAACCAACCATCCTGACGAT	qPCR
		R:GGAGGTCAGCCATTTTTTGGA	
*CPT1A*	NM_001012898.1	F:CTTGCCCTGCAGCTTGCT	qPCR
		R:AGGCCTCGTATGTCAAAGAAATT	
*CPT2*	NM_001031287.2	F:GCCTTCCCTCTTGGCTACCT	qPCR
		R:TCTCAGCAATGCCCACGTATC	
*GAPDH*	NM_204305.1	F:AGAACATCATCCCAGCGT	qPCR
		R:AGCCTTCACTACCCTCTTG	
*CYTB*	YP_009558663.1	F:TCTTACCTGGGTTCTTTCGCC	mtDNA copy number detection
		R:AGTAGTAGGCCGGTGAGGAT	
*ACTB*	NM_205518.1	F:GACCGGCGGGGTTTATATCTT	mtDNA copy number detection
		R:ATTGTCAACAACGAGCGCAG	

**PPARγ, peroxisome proliferator-activated receptor gamma; C/EBPα, CCAAT enhancer binding protein alpha; A-FABP, fatty acid-binding protein 4; ACC, acetyl-CoA carboxylase; FAS: fatty acid synthase; LPL, lipoprotein lipase; ATGL, adipose triglyceride lipase; HSL, hormone-sensitive lipase; PPARα, peroxisome proliferator-activated receptor alpha; CPT1A, carnitine palmitoyltransferase 1A; CPT2, carnitine palmitoyltransferase 2; GAPDH, glyceraldehyde phosphate dehydrogenase; CYTB, cytochrome b; ACTB, actin, beta.**

### Western Blotting

Adipose and liver tissues were lysed with RIPA lysis buffer (1 mM MgCl_2_, 10 mM Tris-HCl at pH 7.4, 1% Triton X-100, 0.1% sodium dodecyl sulfate (SDS), and 1% Non-idet P40 cocktail). The proteins were separated by 5∼12% SDS polyacrylamide gel electrophoresis and transferred to cellulose membranes. The membranes were incubated overnight with the following primary antibodies: GAPDH (Proteintech, United States), ATGL (Proteintech, United States), PPARα (Sangon, Shanghai, China), FABP4 (Sangon, Shanghai, China), and CPT2 (Sangon, Shanghai, China). They were then immunoblotted with secondary antibody (Immunoway, United States). Immunoreactivity was visualized with enhanced chemiluminescence and analyzed with the Quantity One System (BioRad, United States) (Kim et al., 2020; Liang et al., 2020).

### Mitochondrial DNA Copy Number Detection

Fatty acid β-oxidation occurred mainly in mitochondria, and mtDNA copy number is an important index for thermogenesis in adipose tissue (Kavlick, 2019). mtDNA copy number was determined by qPCR as described (Kavlick, 2019; Xia et al., 2020). Briefly, total DNA was isolated from the adipose tissue using column animal genomic DNA purification kit (Sangon, Shanghai, China) according to the manufacturer’s instructions. The relative mtDNA copy number was calculated from the ratio of *CYTB* (mitochondrial encoded gene)/*ACTB* (nuclear encoded gene). DNA was homogenized (100 ng/μl) before qPCR (Y480 real-time PCR detection system, Roche, Switzerland) utilizing SYBR detection (Takara, Dalian, China). Amplification protocol was: 95°C for 30 s, 50 cycles at 95°C to denature, and 60°C for 30 s to anneal. Samples were run in triplicate. All data were normalized by *ACTB* and calculated with the 2^–Δ^ ^Δ^ Ct method (Livak and Schmittgen, 2001).

### Triglyceride and Free Fatty Acid Detection

Blood samples were collected in a vacuum vessel without anticoagulant from jugular vein, and serum was centrifuged at 2,000 rpm for 10 min at 4°C. Triglyceride (TG) and free fatty acid (FFA) contents in serum were determined using commercial kits (Sangon, Shanghai, China) based on kits’ instructions.

### Statistical Analysis

All data were shown as means and standard error (SE). Analysis of variance (ANOVA) was used with SPSS software 18.0 (IBM, Chicago, United States). *P*-values of less than 0.05 were considered to be statistically significant, and the notable differences between groups were identified by Duncan’s multiple comparisons test. Different tissue parameters were divided into embryo and chick periods, their correlations were calculated by simple linear regression, *R*^2^ and *P*-value represent goodness of fit, and slope is significantly non-zero individually.

## Results

### Developmental Changes of Embryo or Chick Growth Parameters and Adipose Contents

**FIGURE 1:**
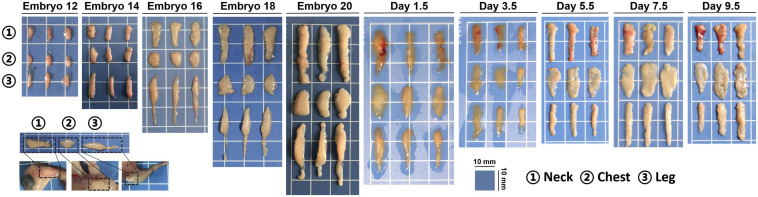
Gross morphology of chick subcutaneous adipose from E12 to D9.5.

As shown in Figure 1, subcutaneous adipose tissue of chicks increased gradually during the embryonic period, but the size of the adipose tissue tended to decrease at posthatch period. Neck white adipose tissue (nWAT) is located outside the jugular vein, chest white adipose tissue (cWAT) is located on the surface of pectoralis, and leg white adipose tissue (lWAT) is located in the anterior of the leg. Most adipose tissues of embryo or chick were subcutaneous adipose tissues and little abdominal adipose at this development stage.

The weight data of adipose and related tissues were collected, and their indexes were calculated. As shown in Supplementary Figure 1, the weight of the embryo gradually increases during the embryonic period, then decreases briefly after hatching and then begins to rise again. The yolk weight was gradually declining, and the liver weight and intestine weight gradually increased from E14 to D9.5.

**FIGURE 2:**
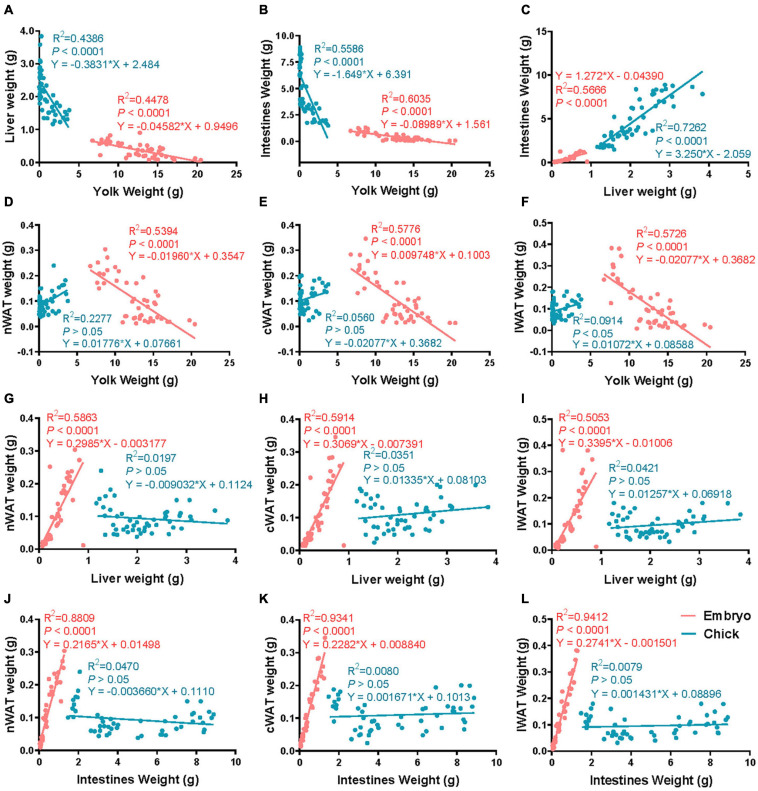
The correlation of subcutaneous fat and related tissues from E12 to D9.5. **(A)** The correlation of liver weight and yolk weight. **(B)** The correlation of intestine weight and yolk weight. **(C)** The correlation of liver weight and liver weight. **(D)** The correlation of nWAT weight and yolk weight. **(E)** The correlation of cWAT weight and yolk weight. **(F)** The correlation of lWAT weight and yolk weight. **(G)** The correlation of nWAT weight and liver weight. **(H)** The correlation of cWAT weight and liver weight. **(I)** The correlation of lWAT weight and liver weight. **(J)** The correlation of nWAT weight and intestine weight. **(K)** The correlation of cWAT weight and intestine weight. **(L)** The correlation of lWAT weight and intestine weight. nWAT, neck white adipose tissue; cWAT, chest white adipose tissue; lWAT, leg white adipose tissue. Pink: embryonic period, blue: posthatch period.

Figures 2A–C shows that there was a strong correlation between yolk weight and liver weight, egg yolk weight and intestinal weight, and liver weight and intestinal weight in both embryo and chick. Figure 2D shows that nWAT weight was strongly correlated with yolk weight of embryo, but not with yolk weight of chick. Figure 2E shows that cWAT weight was strongly correlated with yolk weight in embryo but had no correlation with yolk weight in chick. Figure 2F shows that there was a strong correlation between yolk weight and lWAT weight of embryo but had no correlation in chick. Figure 2G shows that nWAT weight was strongly correlated with liver weight in embryo but had no correlation with liver weight in chick. Figure 2H shows that cWAT weight was strongly correlated with liver weight in embryo, but the cWAT weight had no correlation with liver weight in chick. Figure 2I shows that lWAT weight was strongly correlated with liver weight in embryo, while had no correlation with liver weight in chick. Figure 2J shows that nWAT weight was strongly correlated with intestine weight in embryo but had no correlation with intestine weight in chick. Figure 2K shows that cWAT weight was strongly correlated with intestinal weight in embryo but had no correlation with intestine weight in chick. Figure 2L shows that lWAT weight was strongly correlated with intestine weight in embryo but had no correlation with intestine weight in chick.

### HE Staining and IHC Analysis of Chick Adipose

**FIGURE 3:**
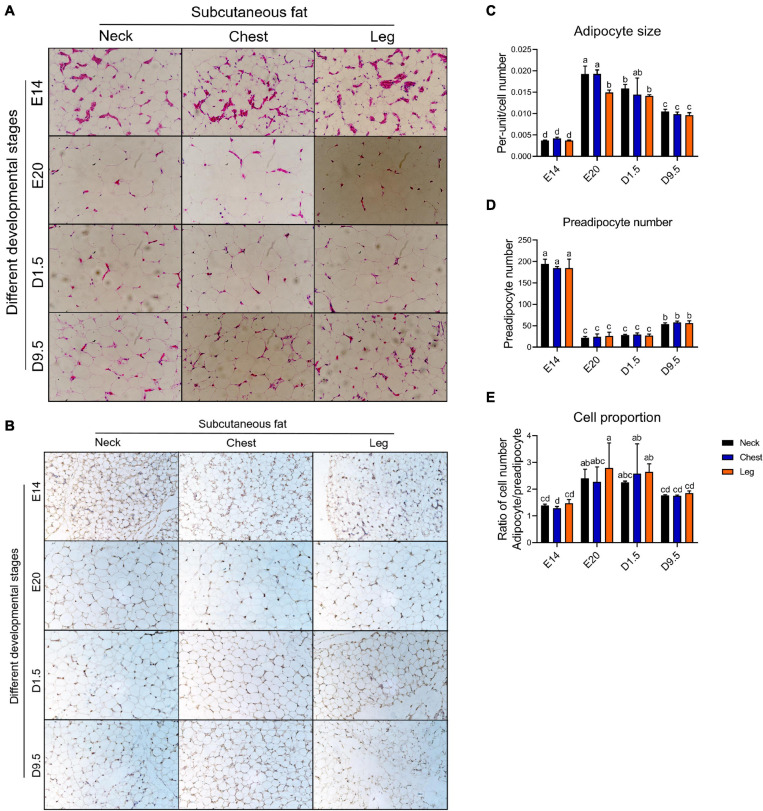
HE and IHC staining analysis of chick adipose. **(A)** HE staining in four development periods. **(B)** IHC staining (pref-1) analysis in four development periods. **(C)** Adipocyte size in four development periods. **(D)** Preadipocyte number in four development periods. **(E)** Ratio of adipocyte to preadipocyte in four developments. HE, hemotoxylin and eosin staining; IHC, immunohistochemical.

Figure 3A shows the HE staining in four development periods. Figure 3B shows that the cell that does not have significant lipid can be marked by pref-1, which was recognized as preadipocyte. Figure 3C shows the adipocyte size in four development periods. In the case of adipocyte size, E20 was significantly larger than D1.5, D9.5, and E14 in the nWAT and cWAT. D1.5 was significantly larger than D9.5 and E14 in the nWAT and cWAT. D9.5 was significantly larger than E14 in the nWAT and cWAT. There was no significant difference in lWAT between E20 and D1.5, but E20 and D1.5 were significantly larger than D9.5 and E14. D9.5 was significantly larger than E14 in the lWAT. Figure 3D shows the preadipocyte number in four development periods. In the case of preadipocyte number, E14 was significantly higher than D9.5, D1.5, and E20. D9.5 was significantly higher than D1.5 and E20. There was no significant difference between E20 and D1.5. Figure 3E shows the ratio of adipocyte to preadipocyte in four developments. E20 was significantly higher than D9.5 and E14, and there was no significant difference from D1.5 in the nWAT and lWAT. E20 was significantly higher than E14, which was not significantly different from D1.5 to D9.5. There were no significant differences between different parts in the same period.

### Expression Patterns of Chick Adipose and Liver During Embryonic and Posthatch Periods

**FIGURE 4:**
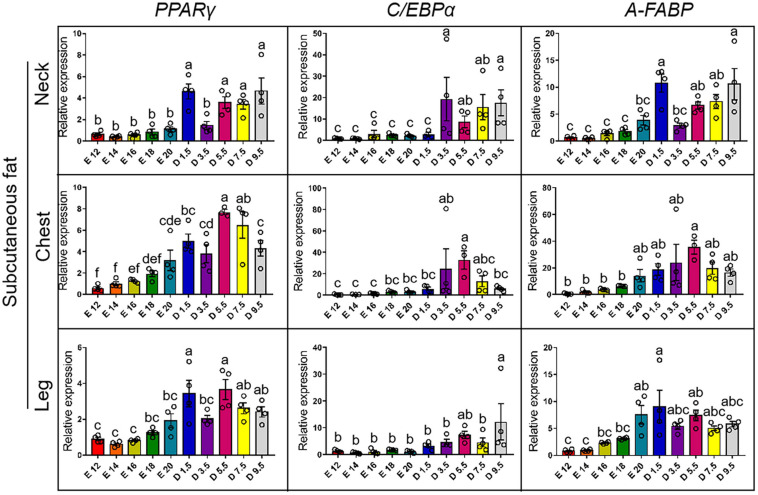
mRNA expression of adipocyte differentiation markers from E12 to D9.5. PPARγ, peroxisome proliferator-activated receptor gamma; C/EBPα, CCAAT enhancer binding protein alpha; A-FABP, fatty acid-binding protein 4.

Figure 4 shows the expression pattern of adipocyte differentiation marker genes from E12 to D9.5. For the expression of *PPAR*γ gene, chicks except D1.5 were significantly higher than embryos in the neck. D5.5 and D7.5 were significantly higher than embryonic stage in the cWAT. D1.5 and D9.5 were significantly higher than E12, E14, E16, and E18 in the cWAT. D1.5 and D5.5 were significantly higher than embryonic stage in the lWAT. For the expression of *C/EBP*α gene, the chicks were significantly higher than embryos in the nWAT, and D5.5 was significantly higher than embryonic stage in the cWAT. D9.5 was the highest in the lWAT and significantly higher than embryonic stage, while D1.5, D3.5, D5.5, and D7.5 were not significantly different from the embryonic stage. For the expression of *A-FABP* (*FABP4*) gene, the expression levels of D1.5 and D9.5 were significantly higher than D7.5, D5.5, D3.5, and embryonic stage in the nWAT. D5.5 was significantly higher than E12, E14, E16, and E18 in the cWAT. D1.5 was the highest in the lWAT and significantly higher than embryonic stage, while D1.5 was not significantly different from other posthatch periods.

**FIGURE 5:**
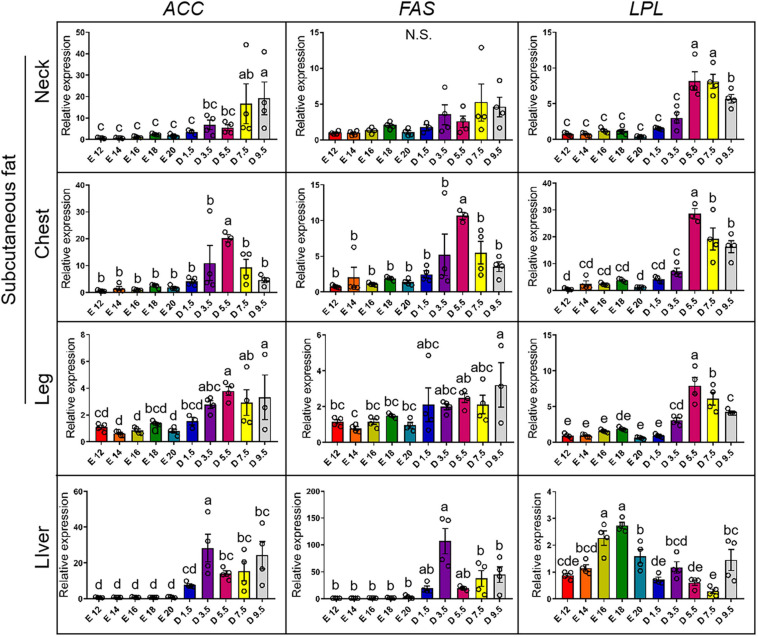
mRNA expression of lipid synthesis genes from E12 to D9.5. ACC, acetyl-CoA carboxylase; FAS, fatty acid synthase; LPL, lipoprotein lipase.

Figure 5 shows the expression pattern of lipid synthesis genes from E12 to D9.5. For the expression of *ACC* gene, D9.5 and D7.5 were significantly higher than embryonic stage in the nWAT. D3.5 and D5.5 were not significantly different from the embryonic stage in the nWAT. D5.5 was the highest in the cWAT and significantly higher than other stages. D5.5 and D9.5 were significantly higher than embryonic stage in the lWAT. D3.5 was significantly higher than other stages in the liver. For the expression of *FAS* gene, there were no significant differences between chicks and embryos in the nWAT. D5.5 was the highest in the cWAT and significantly higher than other stages. D9.5 was the highest in the lWAT and significantly higher than the embryonic stage. D3.5 was the highest in the liver and significantly higher than D7.5, D9.5, and embryonic stage. For the expression of *LPL* gene, D5.5, D7.5, and D9.5 were significantly higher than D3.5 and embryonic stage in the nWAT. D5.5, D7.5, and D9.5 were significantly higher than embryonic stage in the cWAT. D3.5 was significantly higher than E12 and E20 in the cWAT. D5.5 was significantly higher than D7.5, D9.5, D3.5, and embryonic stage in the lWAT. E16 and E18 were significantly higher than other stages, and E20 was significantly higher than D1.5, D5.5, and D7.5 in the liver.

**FIGURE 6:**
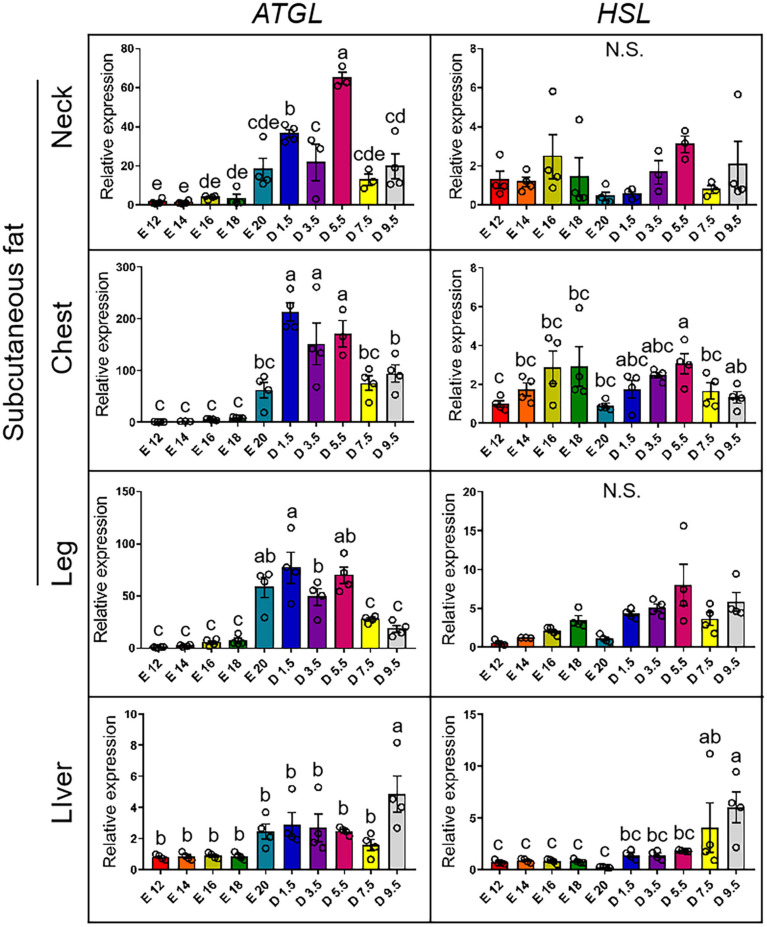
mRNA expression of lipodieresis genes from E12 to D9.5. ATGL, adipose triglyceride lipase; HSL, hormone-sensitive lipase.

Figure 6 shows the expression pattern of lipodieresis genes from E12 to D9.5. In the case of the expression of *ATGL* gene, the chick stages of D1.5, D3.5, and D5.5 were significantly higher than the embryonic stage in the nWAT, cWAT, and lWAT. D9.5 was significantly higher than other stages in the liver. For the expression of *HSL* gene, there was no significant difference in nWAT in different periods. D5.5 in cWAT was significantly higher than the embryonic stage. Except for D5.5, the chick stage was not significantly different with the embryonic stage in the cWAT. The expression level increased from embryonic stage to chick stage, but there was no significant difference between each stage in the lWAT. The chick stage was higher than the embryonic stage in the liver, and D9.5 was significantly higher than other stages.

**FIGURE 7:**
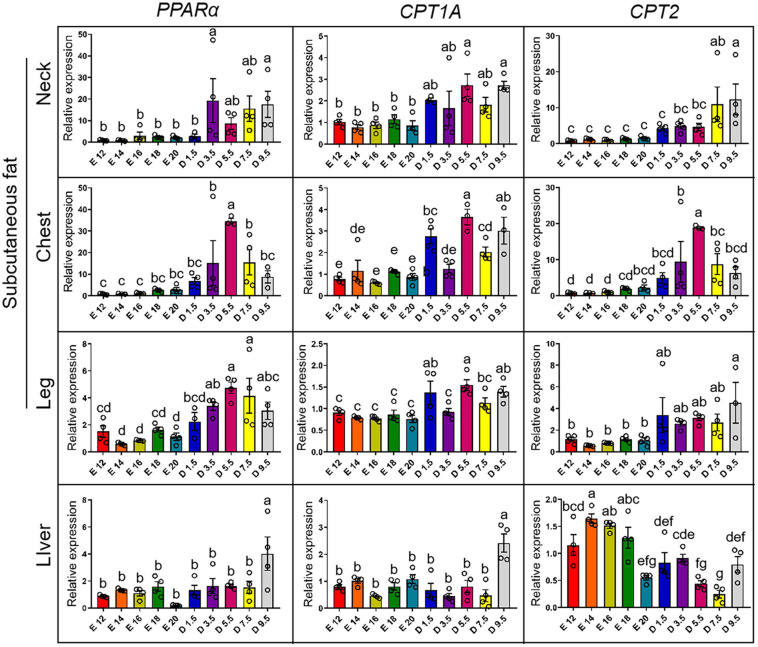
mRNA expression of fatty acid oxidation genes from E12 to D9.5. PPARα, peroxisome proliferator-activated receptor alpha; CPT1A, carnitine palmitoyltransferase 1A; CPT2, carnitine palmitoyltransferase 2.

Figure 7 shows the expression pattern of fatty acid oxidation genes from E12 to D9.5. For the expression of *PPAR*α gene, the chick stages of D3.5 and D9.5 were significantly higher than the embryonic stage in nWAT, cWAT, and lWAT. D9.5 was significantly higher than any other stages in the liver, and there was no significant difference between other periods. For the expression of *CPT1A* gene, the chick stages of D5.5 and D9.5 were significantly higher than the embryonic stage in the nWAT and the chick stage was significantly higher than the embryonic stage in the cWAT but D3.5. D5.5, D1.5, and D9.5 were significantly higher than the embryonic stage in the lWAT. D9.5 was significantly higher than other stages in the liver, and there was no significant difference between other periods. For the expression of *CPT2* gene, the chick stage of D7.5 and D9.5 were significantly higher than the embryonic stage in the nWAT. The chick stage of D5.5 was significantly higher than embryonic stage in the cWAT, and D9.5 was significantly higher than the embryonic stage in the lWAT, but the chick stage was significantly lower than the embryonic stage in the liver and showed a gradual decline from E12 to D9.5.

**FIGURE 8:**
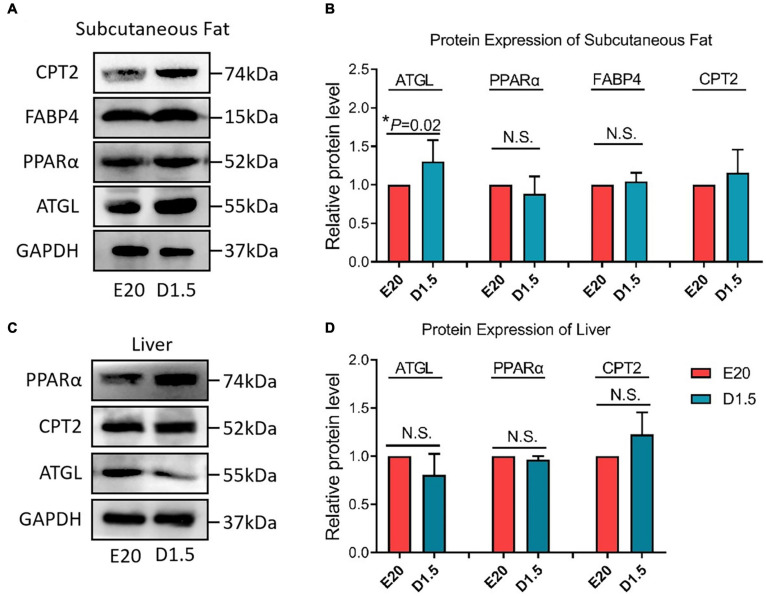
Protein expression of fatty acid oxidation genes in E20 and D1.5. **(A)** Protein expression of subcutaneous adipose in E20 and D1.5. **(B)** Relative protein level expression of subcutaneous adipose in E20 and D1.5. **(C)** Protein expression of liver adipose in E20 and D1.5. **(D)** Relative protein level expression of liver adipose in E20 and D1.5. CPT2, carnitine palmitoyltransferase 2; FABP4, fatty acid binding protein 4; ATGL, adipose triglyceride lipase; GAPDH, glyceraldehyde phosphate dehydrogenase.

Figure 8 shows the key protein expression level between E20 and D1.5. For CPT2, there was no significant difference between embryonic and chick period in WAT and liver. For FABP4, the protein expression in D1.5 was higher than E20 in WAT but had no significant difference in liver. For PPARα, there was no significant difference between embryonic and chick period in WAT and liver. For ATGL, the protein expression in D1.5 was higher than E20 in WAT but had no significant difference in liver.

### Mitochondrial DNA Copy Number of Subcutaneous Adipose Tissue

**FIGURE 9:**
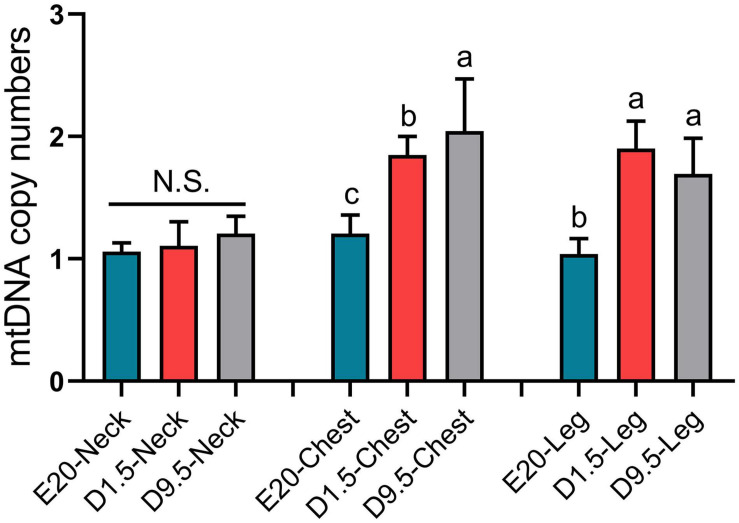
The mitochondrial DNA copy number of subcutaneous fat in E20, D1.5, and D9.5. nWAT, neck white adipose tissue; cWAT, chest white adipose tissue; lWAT, leg white adipose tissue.

To determine whether the massive deposition of subcutaneous adipose tissue in chick embryo was related to thermogenesis, mtDNA copy numbers of E20, D1.5, and D9.5 were measured in nWAT, cWAT, and lWAT. Figure 9 shows that the mtDNA copy number of E20, D1.5, and D9.5 has no significant difference in nWAT; the mtDNA copy number of D1.5 and D9.5 were significantly higher than E14 in cWAT and lWAT.

### TG and FFA Concentration

**FIGURE 10:**
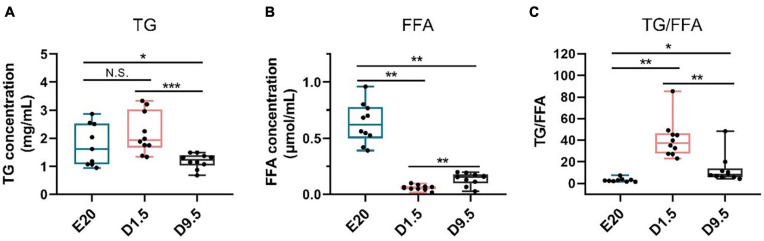
TG and FFA concentration in E20, D1.5, and D9.5. **(A)** TG concentration in E20, D1.5, and D9.5. **(B)** FFA concentration in E20, D1.5, and D9.5. **(C)** Ratio of TG to FFA concentration in E20, D1.5, and D9.5. TG, triglycerides; FFA, free fatty acid. ^∗^*P* < 0.05; ^∗∗^*P* < 0.01; ^***^P < 0.001.

FFA was the main substrate fatty acid β-oxidation. As the hydrolyzate products of TG, FFA could be used in fatty acid β-oxidation for thermogenesis. Figure 10 shows that the TG concentration in E20 and D1.5 was significantly higher than D9.5. The FFA concentration in E20 was higher than D9.5, and D9.5 was higher than D1.5.

## Discussion

The abdominal fat deposition in chicken is closely related to feed conversion efficiency, so it has attracted more attention in poultry production (Chen et al., 2019; Wu et al., 2019). The study-associated subcutaneous adipose tissue has been neglected to some extent, especially during the embryonic period and after hatching (Xiao et al., 2019). In this study, we described a relatively complete process of subcutaneous adipose tissue development in the embryonic period and after hatching. Subcutaneous adipose tissue was the main type of abdominal fat at this stage and was mainly distributed in the neck, chest, and leg. It suggested that a large amount of subcutaneous fat deposition at the embryo period is of important physiological significance to chick. During the embryonic and posthatch period, we found that the body weight, liver, and intestine maintained uninterrupted growth and development, and the yolk weight continued to drop. However, the change of WAT weight presented a different trend; it was increasing at embryonic period and decreasing after hatching (Supplementary Figure 1). The dynamic changes suggested that subcutaneous adipose tissue may play an important physiological role in chicks after hatching.

Obviously, the explanation that subcutaneous adipose tissue is merely an energy supply organ is illogical; the lipid transferred from yolk to subcutaneous adipose tissue caused a partial loss of energy. Yolk is an important tissue in the first few days after the bird was hatched; on account of insufficient energy supply in the digestive system, yolk provides the main energy for body growth and development (Scanes, 2020; Zhu et al., 2020). More importantly, yolk could be absorbed in two different ways before and after hatching. For the hatching period, the yolk was absorbed by yolk sac membrane, but the yolk contents could pass into the intestine *via* the lumen of the yolk stalk in at least newly hatched to 3-day-old chicken (van der Wagt et al., 2020). The presence of subcutaneous adipose tissue could be an energy supply that helps the chicks survive after hatching.

More than 90% of *de novo* lipogenesis in humans and chickens is undertaken by the liver, which was different from rodent (Na et al., 2018; Hicks and Liu, 2021). Therefore, chicken was considered a more suitable model for non-alcoholic fatty liver disease (NAFLD). Enough studies have revealed the details about lipid synthesis, especially the enzymes of lipid synthesis, such as *ACC*, *FAS*, and *LPL* (Mottillo et al., 2017; Seo et al., 2018; Zhang et al., 2019; Bhat et al., 2021). Meanwhile, many studies have concluded that adult chicken fat tissue has no or very weak lipid synthesis ability (Na et al., 2018). In this study, the expression pattern of *ACC*, *FAS*, and *LPL* was described from E14 to D9.5. It was shown that chick has the ability of *de novo* synthesis of FFA, and the ability has been enhanced a few days after hatching. However, the significance of lipid synthesis in subcutaneous adipose tissue for chick is still incomplete.

The expression of *ATGL* in subcutaneous adipose tissue showed that TG was broken down and FFA was released in blood for other tissue uptake. Comparing the expression pattern of *ATGL* in liver, subcutaneous adipose tissue should have a more important role in FFA supplement. Comparing with abdominal adipose tissue, subcutaneous adipose tissue is more avid in absorption of circulating free fatty acids and triglycerides (Ibrahim, 2010). The results of TG and FFA concentration in D1.5 were extraordinary, the TG concentration was high and the FFA concentration was low. The high concentration of TG was continuously inducted into the fat uptake by the liver after hatching; the remaining TG could be absorbed by the subcutaneous adipose tissue. The special phenomenon may be the reason why hepatocyte was full of lipids in new-hatch chickens, which was generally considered a NAFLD animal model (Liu et al., 2018; Peng et al., 2018). It revealed that the function of subcutaneous adipose tissue were absorbing extra TG in blood and releasing FFA from adipocytes. This finding suggested that large amounts of subcutaneous fat deposition could be the inducement of NAFLD and provided a new idea for human treatment of NAFLD.

It was suggested that the subcutaneous adipose tissue could have other important functions during the special period. The subcutaneous adipose tissue weight was not significantly correlated with liver weight and intestine weight (Figure 2). It inferred that the function of adipose tissue may be related to the environmental adaptability of chicks when they were hatched. WAT and BAT are the two main forms of adipose tissue in humans and rodents (Alcalá et al., 2019; Cuevas-Ramos et al., 2019). Thermogenic BAT has never been described in birds or other non-mammalian vertebrates (Mezentseva et al., 2008). Compared with abdominal adipose tissue, the main type of adipose deposition of chicken embryo was subcutaneous adipose tissue (Hicks and Liu, 2021). Interestingly, there were some similarities between the WAT distribution in chickens and the BAT in humans and rodents, and the internal relationship between two adipose tissues needs further study. In this study, it was found that the number of mitochondrial DNA copies in adipose tissue increased significantly within a few days after hatching. The increasing of mitochondrial copy number is an important phenotype of the enhancement of adipose tissue thermogenesis. Due to the loss of *UCP1* gene in chicken, the thermogenesis of subcutaneous adipose tissue depends on *PPAR*α, *PPAR*β, *CPT1A*, and *CPT2* genes (Lee et al., 2016; Martos-Sitcha et al., 2019; Kroon et al., 2020). According to the expression pattern of key factors in thermogenesis, the ability of fatty acid β-oxidation in subcutaneous adipose tissue was increasing in new-hatch period. Although birds do not have BAT, subcutaneous adipose tissue partially functions as brown fat during the period before and after hatching, and its oxidative thermogenesis of fatty acids is greatly improved.

## Conclusion

In this study, the morphology and weight of chick subcutaneous adipose tissue from E14 to D9.5 were characterized, and the expression pattern of adipocyte differentiation, lipid synthesis, lipolysis, and fatty acid β-oxidation from E14 to D9.5 were structured. The results showed that subcutaneous adipose tissue released FFA after hatching and the mitochondrial copy number and fatty acid β-oxidation level significantly increased. It revealed that the function of chick subcutaneous adipose tissue were FFA releasing by lipolysis and thermogenesis by fatty acid β-oxidation. In addition, this finding improved the theory of nutrition supply in embryonic and posthatch period, and might be an important theoretical basis for poultry reproduction.

## Data Availability Statement

The original contributions presented in the study are included in the article/Supplementary Material, further inquiries can be directed to the corresponding author/s.

## Ethics Statement

The animal study was reviewed and approved by The Institutional Animal Care and Use Committee of Northwest A&F University (Permit Number: NWAFAC1019).

## Author Contributions

HZ and MW analyzed the data. HZ wrote the manuscript. HZ, QL, MW, XT, and SW collected the samples. QL performed the qPCR. MW, CJ, XT, and XY reviewed and edited the manuscript. HZ, ZW, and XS designed the experiment. All authors contributed to the interpretation of the results and writing of the manuscript.

## Conflict of Interest

The authors declare that the research was conducted in the absence of any commercial or financial relationships that could be construed as a potential conflict of interest.

## Supplementary Material

Supplementary Figure 1 The weight of chick subcutaneous fat and related tissues from E12 to D9.5. **(A)** Embryo or chick weight, **(B)** yolk weight, **(C)** liver weight, **(D)** intestine weight, **(E)** nWAT weight, **(F)** cWAT weight, and **(G)** lWAT weight. nWAT, neck white adipose tissue; cWAT, chest white adipose tissue, lWAT, leg white adipose tissue.
