# Supplementary figures and images for: Function of Chick Subcutaneous Adipose Tissue During the Embryonic and Posthatch Period
Source: Front Physiol. 2021 Jun 22;12:684426. doi: 10.3389/fphys.2021.684426 (PMC8258255; doi:10.3389/fphys.2021.684426)

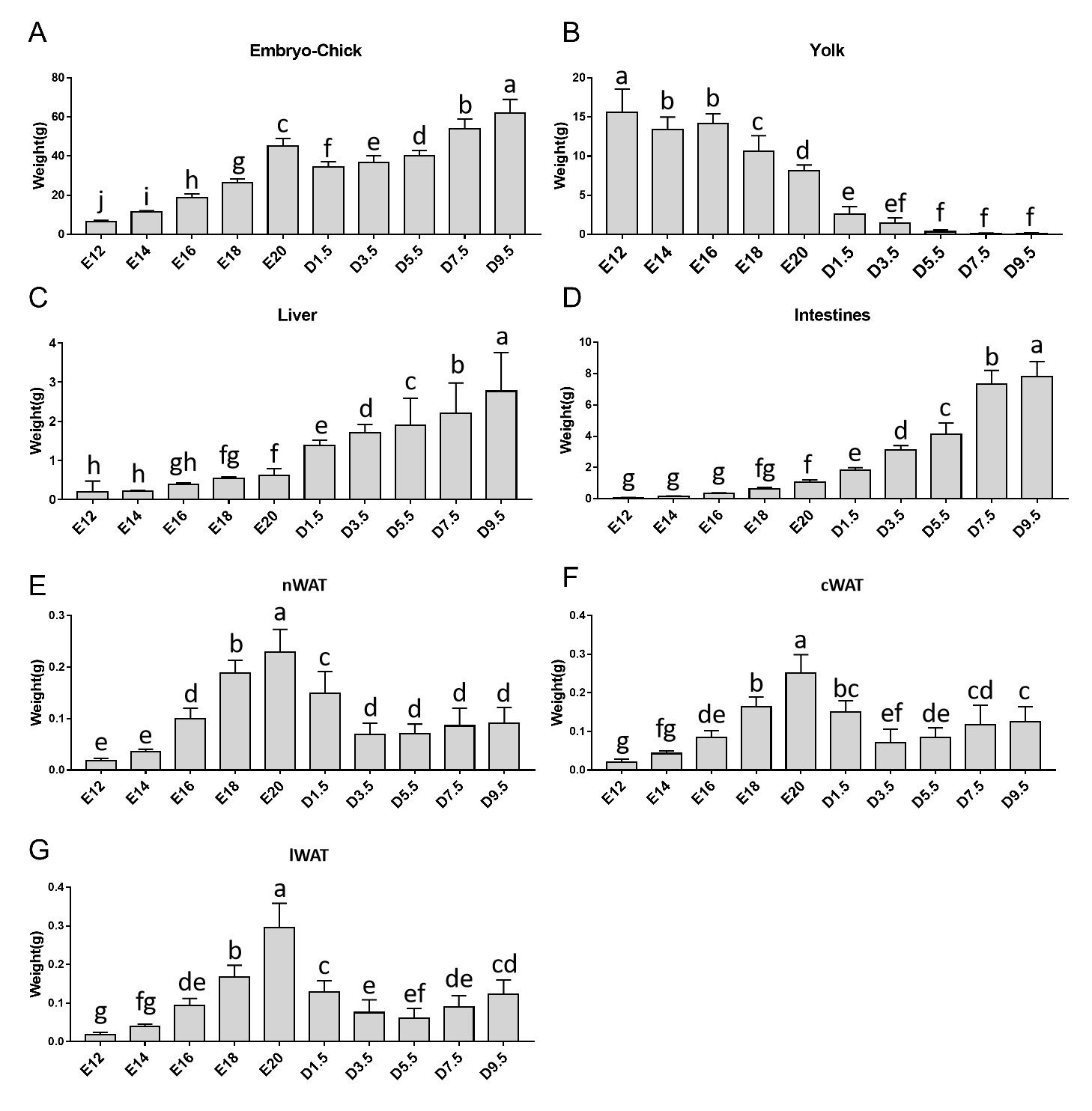

Supplement: Supplementary Figure 1 — The weight of chick subcutaneous fat and related tissues from E12 to D9.5. (A) Embryo or chick weight, (B) yolk weight, (C) liver weight, (D) intestine weight, (E) nWAT weight, (F) cWAT weight, and (G) lWAT weight. nWAT, neck white adipose tissue; cWAT, chest white adipose tissue, lWAT, leg white adipose tissue. [file Image_1.JPEG]
